# Patient-driven second opinions in oncology: a review

**DOI:** 10.1093/oncolo/oyag241

**Published:** 2026-07-14

**Authors:** Mohammad Jahanzeb

**Affiliations:** Department of Integrated Medicine, Charles E. Schmidt College of Medicine, Florida Atlantic University, Boca Raton, FL 33431, United States

**Keywords:** patient-initiated, second opinion, clinical oncology, patients

## Abstract

**Introduction:**

Patient-initiated second opinions have become increasingly common in recent years. However, the frequency, motivations and advantages/disadvantages of this approach remain unclear. This study assessed the incidence and impact of patient-initiated second opinions in oncology patients.

**Methods:**

Literature searches of PubMed and Google Scholar were performed to identify peer-reviewed publications on patient-initiated second opinions in oncology patients published between 06/01/2004 and 06/01/2024. Selected publications evaluated the frequency, patient characteristics and motivating factors of patient-initiated second opinions and their impact on diagnoses, treatment, prognoses and patient satisfaction.

**Results:**

Sixty peer-reviewed publications were included in the analysis. Data showed highly variable rates of second-opinion seeking. Younger age, female gender, higher educational level, advanced disease, socioeconomic status and having health insurance were associated with an increased likelihood of this approach. The most common patient-reported reasons for seeking a second opinion were to gain more information, seek reassurance, access expert guidance, and check the accuracy of proposed treatments. Patient-initiated second opinions led to changes in diagnosis, treatment, or prognosis in up to 69% of cases and a greater likelihood of guideline-compliant recommendations; however, wide variability in reported rates precludes drawing firm conclusions. Patient satisfaction with second-opinion consultations was high and these were deemed helpful and reassuring in most cases.

**Conclusion:**

Increasing numbers of cancer patients are seeking second opinions, and these may have a considerable impact on diagnoses, outcomes and patient satisfaction. Improved patient education and further research regarding the impact of patient-initiated second opinions and barriers to access would be beneficial.

Implications for Practice As more patients seek second opinions, oncology teams should ensure that access pathways are clear, equitable and easy to navigate. Improved communication during initial consultations may reduce unnecessary requests while supporting informed decision-making. Given that second opinions can reveal guideline deviations and lead to clinically meaningful changes in management, incorporating structured opportunities for secondary review may enhance care quality, especially for complex cases and patients with advanced cancers. Efforts to improve patient education and address demographic and geographic barriers will be essential to ensuring that all patients can benefit from this important component of cancer care.

## Introduction

Patients now have unprecedented access to medical information, enabling them to evaluate recommendations, seek alternative counsel, and pursue second opinions.^1–5^ In oncology, where diagnostics and therapies are complex and outcomes critical, patient-initiated second opinions are increasingly common,^6–10^ with most oncologists providing several consultations per month.^1^^,^^5^^,^^6^^,^^11–13^

A patient-driven second opinion can be defined as a patient soliciting independent guidance on diagnosis, prognosis or treatment options from another physician within the same specialty, often with the intention of returning to the original provider.^1^^,^^2^^,^^6^

Reported benefits of second opinions include improved diagnostic accuracy,^6^^,^^14^^,^^15^ greater adherence to guidelines,^14^ more individualized^6^^,^^16^ or de-escalated treatment,^17^ and access to novel diagnostics, technologies and clinical trials,^11^^,^^18^ which is particularly relevant in rare malignancies.^7^ Second opinions may also reduce over- and under-treatment and medical errors.^8^^,^^16^^,^^19^^,^^20^

However, second opinions are not without their challenges, including the potential for diagnostic discrepancies,^8^^,^^9^^,^^20^^,^^21^ treatment delays,^4^^,^^6^^,^^8^^,^^22^ dissatisfaction with the primary clinician,^23^ patient confusion,^6^ and increased clinician workloads.^6^^,^^8^^,^^24^ While second opinions are often sought for reassurance,^6^ up to two-thirds result in changes in diagnosis or treatment.^6^^,^^8^^,^^9^

Despite growing interest in patient-centered care, the evidence base for patient-initiated second opinions remains limited.^1^^,^^9^^,^^16^ Only 5 systematic reviews on this topic were published between 2004 and 2024,^6–10^ of which 3 focused on oncology (Table S1).^6^^,^^7^^,^^10^ Most studies are in breast cancer,^25^ with substantially fewer in other tumor types, particularly rare cancers.^26^ The literature remains fragmented, with inconsistent prevalence estimates, limited reporting of equity factors (eg, socioeconomic status, insurance, ethnicity), and little exploration of long-term outcomes.^6^^,^^8^^,^^9^^,^^27–29^

This review focuses on patient-initiated second opinions in oncology, examining which patients seek them, their motivations, and the resulting clinical impact.

## Materials and methods

Structured searches of PubMed and Google Scholar were conducted to identify peer-reviewed manuscripts, posters, and congress abstracts published between June 1, 2004 and June 1, 2024 that examined patient-initiated second opinions in oncology. Searches combined patient-referral terms (eg, *second opinion*/s*econd medical opinion*, *patient request*, *self-initiated*), tumor-related terms (*cancer*, *oncology*, *tumor*, *neoplasm*, *sarcoma*, *hematology*), and publication-type descriptors (*meta-analysis*, *systematic review*, *review*, *observational or clinical study*, *interview*, *survey*, *retrospective review*). Exclusion criteria comprised non-patient-initiated consultations; non-English or non-peer-reviewed publications; case reports or case series; studies focusing on non-oncology or palliative-care populations; and ethics consultations, guidelines, editorials, commentaries, or unpublished material. Studies from other medical specialties were included only if oncology-specific outcomes were reported separately. Only papers explicitly addressing second opinions were included; citations without such content and pathology studies describing physician-initiated reviews were excluded. Forward and backward citation searches of eligible papers were performed to ensure completeness.

The review was guided by predefined questions examining: the proportion of patients who request a second opinion; the clinical and demographic characteristics of those who do so; their motivations; the independence, guideline concordance, and alignment of second-opinion recommendations with initial recommendations; patient satisfaction with second-opinion consultations; and potential inequities in access to or outcomes from second opinions based on patient-related factors.

## Results

A total of 127 publications were identified through database and citation searches. Sixty-seven publications were excluded: second opinions were not patient-initiated (*n* = 27); non-oncology focus (*n* = 15); out of scope (*n* = 10); editorials/commentaries/magazine articles (*n* = 9); non-English (*n* = 3); or published before 2004 (*n* = 3). Sixty publications met the inclusion criteria and were utilized in the analysis.

Most included studies were quantitative cohort/cross-sectional surveys (*n* = 22), retrospective chart/imaging reviews (*n* = 16), or qualitative interviews/focus groups (*n* = 8). Additional designs included policy/health service evaluations (*n* = 6), systematic reviews/meta-analyses (*n* = 5), longitudinal mixed-methods studies (*n* = 2), and 1 conceptual paper (Table S1). Patient selection procedures varied by study methodology.

### Proportion of patients requesting a second opinion

Reported rates of second-opinion seeking among patients with cancer varied widely, ranging from 6.5% to 42% across studies.^1^^,^^4^^,^^10^^,^^13^^,^^25–33^ Questionnaire-based surveys conducted in Australia, Germany, Hong Kong, the Netherlands and the US illustrate this variation, with rates of 6.5% (123/1892),^1^ 16.1% (57/355),^31^ 23% (427/1856),^26^ 33% (17/52),^13^ 34% (36/106),^30^ 40% (950/2365),^32^ and 42% (80/191)^33^ (Table 1).

**Table 1:** Variables associated with second-opinion seeking in oncology patients (presented alphabetically by variable).

Variable	Cancer type	Country	Summary of evidence
**Age**	Breast,^12^^,^^36^ colorectal,^34^^,^^36^ prostate,^32^^,^^36^ mixed^1^^,^^35^	Australia,^12^ Germany,^1^ India,^35^ ,^12^ United States^32^^,^^34^^,^^36^	Younger patients were more likely to seek SOs^1^^,^^12^^,^^32^^,^^34–36^ SO-seeking was disproportionate to cancer incidence in the 17-44-year age group^35^
**Cancer type**	Breast,^4^ gynecologic,^33^ prostate,^32^ rare,^26^ mixed^1^^,^^13^^,^^30^	Australia,^1^^,^^13^ Germany,^30^Hong Kong,^33^ the Netherlands,^26^ United States^4^^,^^32^	Rates of SO seeking varied by malignancy,^1^^,^^4^^,^^13^^,^^26^^,^^30^^,^^32^^,^^33^ from 23% in rare cancers^26^ to 40% in prostate^32^ and 42% in gynecologic cancers^33^ Rare cancers showed mixed patterns compared with common cancers^26^
**Disease stage**	Breast,^4^^,^^43^ bladder,^44^ colorectal,^34^ gynecologic,^33^ prostate,^44^ mixed^31^^,^^35^^,^^44^	Australia,^31^ France,^44^ Hong Kong,^33^ India,^35^ United States^4^^,^^34^^,^^43^	Advanced/recurrent disease was associated with higher rates of SO seeking^9^^,^^33–35^ ^44^ Patients with early‑stage breast cancer were generally less likely to seek SOs^43^ One study showed higher rates of SO seeking in patients with lower pathologic tumor stage^4^; another showed no impact of disease stage^31^
**Educational attainment**	Breast,^36^^,^^38^ colorectal,^36^^,^^38^ gynecologic,^33^ lung,^38^ prostate,^32^^,^^36^ mixed^1^^,^^6^^,^^8^^,^^31^	Australia,^1^^,^^31^ Hong Kong,^33^ ^8^ international^6^^,^^8^ United States^32^^,^^36–38^	Patients with higher educational attainment were generally more likely to seek a second opinion^1^^,^^6^^,^^8^^,^^32^^,^^33^^,^^36–38^ This association was not consistent across all studies^6^^,^^31^
**Gender**	Mixed^1^^,^^6^^,^^8^^,^^9^^,^^30^	Australia,^1^ Germany,^30^International, ^6^^,^^8^^,^^9^	SO seekers were more often female^1^^,^^6^^,^^8^^,^^9^ Some studies showed no gender-related differences^6^^,^^8^ or contradictory findings^30^
**Geographic factors**	Breast,^37^ mixed^35^	India,^35^ United States^37^	Place of residence (metropolitan vs rural) and availability of a specialist center influenced rates of SO seeking^35^^,^^37^ Mixed findings were observed across countries^35^^,^^37^
**Health insurance**	Lung/colorectal,^38^ prostate,^32^ mixed^31^	Australia,^31^ United States^32^^,^^38^	Insurance coverage was an independent predictor of SO seeking^31^^,^^32^ In one study, health insurance was reported to double the odds of seeking an SO^31^ Another study reported that the type of insurance did not impact the likelihood of self-referral^38^
**Healthcare system factors**	Breast^37^	United States^37^	Higher SO seeking was reported when the diagnosis came from primary care or community hospitals vs specialist centers^37^
**Race/ethnicity**	Breast,^4^^,^^42^ lung/colorectal,^34^^,^^38^ prostate^41^	United States^4^^,^^34^^,^^38^^,^^41^^,^^42^	Lower rates of SO seeking were reported among Black vs White patients,^38^^,^^41^ and Black patients showed greater mistrust of clinicians^41^ Communication barriers exist for non-English-speaking patients and few have access to interpreters^42^ Some studies found no significant associations between SO seeking and race^4^^,^^34^
**Socioeconomic status**	Breast,^22^^,^^37^^,^^38^ lung/colorectal,^38^ mixed^8^^,^^40^	Israel,^8^^,^^40^ , United States^22^^,^^37^^,^^38^	Across multinational and country‑specific studies, higher SES increased the likelihood of SO seeking^8^^,^^22^^,^^37^^,^^38^^,^^40^ In one study, low income increased time to treatment^22^
**Treatment modality**	Gynecologic,^33^ mixed^39^	Hong Kong,^33^ Japan^39^	Radiotherapy was associated with higher rates of SO seeking than surgery^33^^,^^39^

Abbreviations: SES, socioeconomic status; SO, second opinion.

### Characteristics of patients requesting a second opinion

Across studies, second-opinion seeking was typically more common among younger, female patients,^1^^,^^6^^,^^8^^,^^9^^,^^12^^,^^32^^,^^34–36^ individuals with higher educational attainment^1^^,^^6^^,^^8^^,^^32^^,^^33^^,^^36–39^ or socioeconomic status,^8^^,^^22^^,^^37^^,^^38^^,^^40^ and those with health insurance.^31^^,^^32^^,^^38^ However, these associations were not consistent, with several studies reporting contradictory findings.^4^^,^^6^^,^^30^^,^^31^ A number of publications reported racial and linguistic disparities, although findings were not uniform.^4^^,^^34^^,^^38^^,^^41^^,^^42^ Disease stage,^4^^,^^31^^,^^33–35^^,^^43^^,^^44^ tumor type,^1^^,^^3^^,^^4^^,^^26^^,^^30^^,^^33^ and treatment modality^33^^,^^39^ also influenced second-opinion requests, with higher rates observed in some advanced/recurrent^9^^,^^33–35^ and complex cases,^44^ and among patients receiving radiotherapy.^33^^,^^39^ Geographic and healthcare-system factors, including urban versus non-urban residence, referral pathways, and access to specialist centers, further shaped patterns of second-opinion seeking.^35^^,^^37^ These variables and the supporting evidence are summarized in Table  1.

### Patient-reported reasons for requesting a second opinion

Patients most commonly sought a second opinion to seek reassurance about or confirm the correctness of the diagnosis and proposed treatment plan^1^^,^^13^^,^^27^^,^^30–32^^,^^40^^,^^45–50^; to access expert guidance^27^^,^^30^^,^^32^^,^^45^^,^^46^^,^^51^; or to obtain additional information.^1^^,^^27^^,^^30^^,^^32^^,^^46^^,^^49^^,^^51^ Dissatisfaction with or poor understanding of the initial information provided^1^^,^^12^^,^^30^^,^^51^; uncertainty about their situation^27^^,^^30^^,^^31^^,^^47^^,^^51^; and dissatisfaction with or lack of trust in the first clinician^12^^,^^29^^,^^31^^,^^46^^,^^51^ also contributed. Clinical trial opportunities were reported less frequently as reasons for seeking a second opinion.^31^^,^^45^^,^^49^^,^^51^ These motivations are summarized in Table  2.

**Table 2:** Motivations most commonly reported by oncology patients for seeking a second opinion (ranked from highest to lowest frequency).

Patient-reported motivations	Frequency (% of surveyed patients)
**Seeking confirmation or reassurance about the proposed diagnosis or treatment plan^1^^,^^13^^,^^27^^,^^30–32^^,^^40^^,^^45–50^**	49-81^1^^,^^30^^,^^31^^,^^46^
**Desire to consult the “best doctor,” access expert guidance, or understand what other specialists would advise^27^^,^^30^^,^^32^^,^^45^^,^^46^^,^^51^**	46-70^30^^,^^32^^,^^45^^,^^46^^,^^51^
**Seeking additional information about the diagnosis, prognosis or available treatment options^1^^,^^27^^,^^30^^,^^32^^,^^46^^,^^49^^,^^51^**	24-75^1,^^30^^,^^32^^,^^46^^,^^49^^,^^51^
**Dissatisfaction with or poor understanding of the information provided initially^1^^,^^12^^,^^30^^,^^51^**	>15-31^1,^^30^^,^^51^
**Dissatisfaction with, poor relationship with, or lack of trust in the initial treating clinician^12^^,^^29^^,^^31^^,^^46^^,^^51^**	10-24^29^^,^^31^^,^^46^^,^^51^
**Uncertainty or confusion regarding their clinical situation and the information received^27^^,^^30^^,^^31^^,^^47^^,^^51^**	7-16^31^^,^^51^
**Clinical trial opportunities^31^^,^^45^^,^^49^^,^^51^**	3-10^31^^,^^45^^,^^49^^,^^51^

### Are second-opinion recommendations independent, guideline-compliant and do they align with first-opinion recommendations?

Independence and concordance between first- and second-opinion recommendations displayed considerable variation across studies (Table  3).^10^^,^^17^^,^^25^^,^^29^^,^^44^^,^^50^^,^^52–57^ First-opinion recommendations, inter-physician dynamics, and the public nature of second opinions were all reported to influence outcomes.^50^^,^^52^

**Table 3:** Independence, guideline compliance, and concordance between first and second opinions in self-referred oncology patients.

Country	Study type	Population, *N*	Key findings
**Independence of second-opinion recommendations**			
**The Netherlands^52^**	Qualitative interview	Oncologists/hematologists (*N* = 26)	SO clinicians avoided reporting minor discrepancies between first and second opinions to protect relationships with referring physicians When first and second opinions aligned, SO clinicians felt constrained by the risk of diminishing patients’ hope
**Guideline compliance and quality of first- and second-opinion recommendations**			
**Germany^14^**		Oncology patients, mixed (*N* = 584)	Among initial consultations, 54.5% were fully guideline-compliant, 13.2% were partially compliant and 6.8% were non-compliant Without an SO, ≥20% of cases would have lacked full guideline-based recommendations
**Germany^58^**	Retrospective, observational	Patients with breast/gynecologic cancers (*N* = 164)	34.8% of primary consultations were not guideline-compliant SO consultations optimized care in 56.7% of cases, often via changes in systemic therapy (30.5%)
**Levels of concordance between first and second opinions**			
**^10^**	Systematic review	Oncology patients, mixed	SO review resulted in changes in diagnosis, treatment recommendations or prognosis in 12%-69% of cases
**The Netherlands^29^**	Longitudinal mixed-methods	Oncology/hematology specialists (*N* = 24)	Most feedback from SO clinicians regarding referring clinicians was positive: 73.8% confirmed/defended the referring oncologist 51.3% confirmed/supported their actions
**United States^17^**	Retrospective chart/imaging review	Oncology patients, mixed (*N* = 120)	35% of patients experienced clinically meaningful changes in treatment following SO, which improved prognoses and/or morbidities
**United States^55^**	Policy/health services/economic evaluation	Patients across medical specialties seeking SOs (*N* = 6791); medical oncology/hematology (*n* = 588)	In the medical oncology/hematology patient cohort, SOs resulted in changes in diagnosis in 5.1% of patients and treatment in 27.0% of patients These rates were lower than for the overall population (14.8% and 37.4%, respectively)
**Discrepancies between first and second opinions by cancer type: breast cancer**			
**The Netherlands^25^**	Retrospective chart/imaging review	*N* = 591	Major discrepancies between first and second opinions were reported in 29.1% of cases, mainly relating to primary treatment, type of first surgery, and use of IBR 22.8% of patients were switched between surgery and neoadjuvant therapy
**United States^56^**	Retrospective chart/imaging review	*N* = 1970	Clinically significant discrepancies (affecting patient care) between initial and SO consultations were reported in 11.47% of cases
**United States^53^**	Retrospective chart/imaging review	*N* = 147 (176 lesions)	Among patients without a diagnosis of breast cancer who self-referred for an SO following imaging, SO was discordant with the first opinion in 47% of lesions 35% of cancers diagnosed after SO review represented malignancies not initially detected during first review
**Discrepancies between first and second opinions by cancer type: urologic cancers**			
**France^44^**		Patients across medical specialties (*N* = 1552), including bladder (*n* = 12) and prostate (*n* = 46) cancers	Rates of divergence between first and second opinions were 33% in patients with bladder cancer and 35% in those with prostate cancer Complex cases were associated with significantly higher risks of divergence between first and second opinions (OR 2.78)
**United States^54^**	Cohort/cross-sectional survey (quantitative)	Survey of patients with prostate cancer (*N* = 238) and their urologists	Urologists recommended fewer treatment options during SOs Recommendations for surgery increased from 71% to 91% from first to second opinions, including for low-risk disease
**Discrepancies between first and second opinions by cancer type: lung cancer**			
**The Netherlands^57^**	Retrospective chart/imaging review	Patients with lung cancer (*N* = 188)	Discrepancies were identified between initial and second opinions in diagnosis (9%), staging (13%) and therapy recommendations (37%) Discrepancies had a potentially major impact on outcomes in 28% of cases, especially in patients with advanced disease
**Impact of who initiates the second opinion**			
**United States^69^**	Retrospective chart/imaging review	Patients with prostate cancer (*N* = 684)	Diagnostic changes were more frequent when SOs were urologist- vs patient-initiated (41.4% vs 25.0%; *P* < .0001)

Abbreviations: IBR, immediate breast reconstruction; OR, odds ratio; SO, second opinion.

Guideline compliance was inconsistent. In breast and gynecologic cancers, 35% of primary consultations were non-compliant, while second opinions optimized care in 57%.^58^ In a mixed oncology cohort, at least 20% of patients would not have received fully guideline-based recommendations without a second opinion.^14^

Reported rates of discrepancies between first- and second-opinion consultations varied widely between analyses (5%-69%).^10^^,^^17^^,^^25^^,^^29^^,^^44^^,^^53–57^ Second opinions resulted in clinically meaningful changes in diagnosis, staging and, particularly, treatment recommendations across multiple tumor types (breast,^25^^,^^53^^,^^56^ lung,^57^ and prostate/bladder^44^^,^^54^ cancers). Alterations in patient management resulting from second opinion consultations were shown to affect outcomes, particularly in patients with advanced disease.^17^^,^^57^ Impacts varied by tumor type, resulting in shifts toward surgical management in urologic cancers,^54^ and changes in treatment sequencing in breast cancer.^25^

### Patient satisfaction with second-opinion consultations

Across multiple settings, patient satisfaction with second-opinion consultations was consistently high.^10^^,^^13^^,^^30^^,^^46^ In Australia (*N* = 52)^13^ and Germany (*N* = 106^30^ and *N* = 164^46^) between 79% and 94% of patients described second opinions as helpful and reassuring, and 90% of patients in one study reported feeling better informed than after their initial consultation (*P* < .0001).^46^ These gains were linked to increased reassurance and improved communication.^13^^,^^46^ Findings from the Netherlands (*N* = 70)^51^ showed a more nuanced pattern; although 66% of patients with advanced disease expected different outcomes, most ultimately found the second opinion to be either “largely” (56%) or “completely” (33%) consistent with the first. This alignment reduced uncertainty but did not lessen anxiety.^51^

Patient satisfaction with second opinions also varied depending on subsequent care pathways. In a prospective study of 69 patients and 24 oncologists,^23^ those referred back to their original institution after receiving a second opinion were significantly less satisfied than those who were transferred to the second-opinion center (*P* = .028). Educational background also appeared to influence confidence; at an Australian cancer center (*N* = 77),^1^ 67% of patients with secondary education felt confident following a second opinion, compared with 36% of those with tertiary education (*P* = .046).

### Are there inequities in access to or outcomes from a second opinion based on patient-related factors?

Evidence across multiple settings indicated potential inequities in access to second opinions relating to insurance status/income, ethnicity, education/health literacy, and geography (Table 1).^1^^,^^4^^,^^12^^,^^26^^,^^31–34^^,^^37^^,^^38^^,^^41–43^

Uninsured oncology patients were substantially less likely than insured patients to obtain a second opinion in both Australian (*N* = 355)^31^ and US (*N* = 2365)^32^ cohorts. Further inequities were observed among women with ductal carcinoma *in situ* (*N* = 710), where Spanish-speaking Latinas accessed second opinions less often than White or English-speaking Latina women.^42^ Several studies also reported lower rates of second-opinion seeking among individuals with lower versus higher educational attainment,^1^^,^^32^^,^^33^^,^^36–38^ and among Black compared with White patients,^38^^,^^41^ alongside greater mistrust of clinicians among Black patients.^41^ Geographic variation contributed as well; Dutch patients with rare cancers (*n* = 1856) were significantly more willing than those with common cancers (*n* = 5487) to travel further for specialist input (55% vs. 47%, *P* < .001), highlighting uneven access to specialist services.^26^

## Summary

In this analysis, multiple studies showed that second opinions can meaningfully alter diagnoses, treatment recommendations, or prognoses, with reported changes in up to 69% of cases.^10^^,^^17^^,^^20^^,^^25^^,^^44^^,^^56^^,^^57^ Despite this potential impact, published rates of second-opinion seeking varied widely.^1^^,^^4^^,^^9^^,^^24–33^ This reflects heterogeneity in tumor type,^1^^,^^3^^,^^4^^,^^26^^,^^30^^,^^32^^,^^33^ disease stage,^4^^,^^31^^,^^33–35^^,^^43^^,^^44^ patient characteristics,^1^^,^^6^^,^^8^^,^^9^^,^^12^^,^^18^^,^^22^^,^^31–40^ treatment modality,^33^^,^^39^ and geographic setting.^35^^,^^37^

Patterns of second-opinion use differed markedly across healthcare systems.^6^^,^^10^^,^^24^ In the US^17^^,^^32^^,^^34^^,^^38^^,^^43^^,^^48^ and Australia,^1^^,^^13^^,^^50^ second opinions were often associated with changes in treatment plans or improvements in guideline adherence, whereas in continental Europe they were more commonly embedded in structured cancer-center programs focused on quality assurance.^6^^,^^7^^,^^12^^,^^14^^,^^23^^,^^46^^,^^58–61^ In Israel, second opinions were highly utilized and examined through a health-policy lens,^8^^,^^16^^,^^19^^,^^24^ while in the Asia-Pacific region they were more strongly shaped by cultural norms, with less emphasis on clinical discordance.^33^^,^^39^^,^^62^

Data showed racial and socioeconomic inequities in access to and outcomes from second opinions.^6^^,^^8^^,^^22^^,^^31^^,^^32^^,^^37^^,^^38^^,^^40^ Patients with rare cancers appeared more willing to travel further for specialist care than those with common cancers,^26^ while low income and African American or Latin American ethnicities were associated with greater treatment delays following second opinions in breast cancer.^22^ UK data suggest that Caribbean, African and Asian women with breast or ovarian cancer face higher odds of late-stage diagnosis than White women,^63^ which may deter second-opinion seeking. Low socioeconomic status and lack of health insurance also restrict access, disproportionately affecting minority groups.^64^ Across studies, higher socioeconomic status,^6^^,^^8^^,^^22^^,^^37^^,^^38^^,^^40^ and insurance coverage^31^^,^^32^^,^^38^ consistently predicted second-opinion seeking. However, most of the studies in this analysis derived from the US, limiting generalizability to universal healthcare systems.

Reported incidences of second-opinion seeking appeared to be substantially lower than expressed patient intent; although 78% of surveyed members of the German public^59^ and 79% of general hospital patients in Japan^62^ reported willingness to seek a second opinion for a cancer diagnosis, only 1.6% of >2.6 million Japanese cancer patients did so, possibly due to consultation fees and cultural deference.^65^ Low reported frequencies of second-opinion seeking may reflect limited patient awareness; indeed, in another German study, 94% of patient respondents expressed a desire for a second opinion but only 20% knew it was available.^66^ Barriers to second-opinion seeking among cancer patients include shock following diagnosis; time pressures; information overload; and fear of harming the patient–clinician relationship.^67^ Limited patient awareness^66^ and patient-related factors further contribute.^1^^,^^6^^,^^9^^,^^12^^,^^68^ These findings highlight the need for patient education on the availability, purpose and implications of second opinions, and how to interpret potentially conflicting information.

This review has several limitations. It was not systematic, lacked quantitative analysis, and drew on studies with substantial methodological heterogeneity, many of which relied on self-reported data with variable and inconsistent findings. As a result, evidence quality and comparability were limited, and few studies examined inequities in access to, or the cost-effectiveness of, second opinions. Despite these constraints, this review offers a broad overview of patient-initiated second opinions in oncology, outlining key trends, challenges, evidence gaps, and opportunities for advancement, including patient education.

Further research is needed to define the incidence and impact of second opinions in oncology. Key priorities include inequity analyses, cost-effectiveness evaluations, and clarification of the patient groups who derive the greatest benefit. Broader use of collaborative decision-making models (eg, tumor boards) and second-opinion programs could reduce unnecessary consultations and improve outcomes capture, equity access, and reporting consistency. Emerging artificial intelligence tools may also contribute. Together, these strategies could strengthen the evidence base and optimize the role of second opinions in oncology care.

## Conclusions

Second opinions are sought by increasing numbers of oncology patients and, despite wide variation across populations and healthcare systems, consistently provide meaningful clinical and informational benefit. Use is patterned by demographic, socioeconomic and disease-related factors, with persistent inequities linked to ethnicity, language, education, income, insurance status and geography. Patients most often seek additional information, reassurance and expert confirmation. In turn, second opinions frequently improve guideline adherence and lead to clinically relevant changes in diagnosis, staging or treatment recommendations. Patient satisfaction is generally high, although influenced by care pathways and patient characteristics. Overall, these findings highlight the value of second opinions in enhancing care quality while underscoring the need to improve patient education and address other barriers to equitable access.

## Supplementary Material

oyag241_Supplementary_Data

## Data Availability

The data presented in this study are available on reasonable request from the author.
