# Supplementary material for: Patient-driven second opinions in oncology: a review
Source: Oncologist. 2026 Jul 14;31(8):oyag241. doi: 10.1093/oncolo/oyag241 (PMC13399442; doi:10.1093/oncolo/oyag241)
Supplement: oyag241_Supplementary_Data [file oyag241_supplementary_data.docx]

**Table S1.** Overview of the characteristics of the included studies.

|  | **Category** | | **Subcategory** | **Studies, N** | **First author, year** |
| --- | --- | --- | --- | --- | --- |
| 1 | | Tumor type | Bladder | 1 | Sanchez, 2021^44^ |
|  |  |  | Breast | 19 | Beer, 2022;^4^ Blazek, 2021;^22^ Cecon, 2019;^68^ Coffey, 2018;^53^ Coffey, 2017;^S3^ Garcia, 2018;^20^ Groß, 2017;^12^ Heeg, 2019;^25^ Khazai, 2015;^56^ Kurian, 2017;^43^ Loehberg, 2020;^46^ Lund, 2009;^37^ Lux, 2020;^58^ McCranie, 2024;^18^ Moumjid, 2007;^2^ Nagler, 2010;^36^ Oskay-Özcelik, 2007;^66^ Parikh, 2013;^42^ Weinfurtner, 2018^S1^ |
|  |  |  | Colorectal | 5 | Chang, 2013;^11^ de Roo, 2020;^34^ McCranie, 2024;^18^ Nagler, 2010;^36^ Pollack, 2015^38^ |
|  |  |  | Gynecologic | 3 | Loehberg, 2020;^46^ Lux, 2020;^58^ Tam, 2005^33^ |
|  |  |  | Head and neck | 1 | Lysack, 2013^52^ |
|  |  |  | Hematologic | 1 | Goldman, 2009^27^ |
|  |  |  | Lung | 3 | Al Achkar, 2022;^45^ Pollack, 2015;^38^ Schook, 2014^57^ |
|  |  |  | Pancreatic | 1 | McCranie, 2024^18^ |
|  |  |  | Prostate | 8 | Chan & Epstein, 2005;^69^ Denberg, 2006;^48^ Hillen, 2017b;^47^ Nagler, 2010;^36^ Radhakrishnan, 2015;^32^ Ramsey, 2011;^54^ Sanchez, 2021;^44^ Xu, 2011^41^ |
|  |  |  | Rare | 3 | de Heus, 2021;^26^ Lipitz-Snyderman, 2022;^17^ Maruzzo, 2021^7^ |
|  |  |  | Solid tumors | 8 | Hillen, 2023;^29^ Lehmann, 2020^51^/Lehmann, 2021;^23^* Lipitz-Snyderman, 2022;^17^ Palmieri, 2017;^61^ Payne, 2014;^9‡^ Rajan, 2021;^49^ Schulmeyer, 2023;^14^ van der Velden, 2021^60^ |
|  |  |  | All types | 13 | Fuchs, 2017;^30^ Goud, 2021;^35^ Greenfield, 2021;^8‡^ Hillen, 2017a;^6^ Maruzzo, 2021;^7^ Meyer, 2015;^55^ Minamitani, 2021;^39^ Mordechai, 2015;^40^ Olver, 2020;^31^ Peier-Ruser, 2018;^67^ Philip, 2010;^13^ Ruetters, 2016;^10^ Tattersall, 2009^1^ |

| 2 | | Tumor stage | Pre-diagnosis | 3 | Chan & Epstein, 2005;^69^ Coffey, 2018;^53^ Coffey, 2017^S3^ |
| --- | --- | --- | --- | --- | --- |
|  |  |  | Newly diagnosed | 8 | Cecon, 2019;^68^ Chan & Epstein, 2005;^69^ Chang, 2013;^11^ Denberg, 2006;^48^ Groß, 2017;^12^ Kurian, 2017;^43^ Lipitz-Snyderman, 2022;^17^ Ramsey, 2011^54^ |
|  |  |  | Mixed stage | 31 | Al Achkar, 2022;^45^ Blazek, 2021;^22^ de Roo, 2020;^34^ Goud, 2021;^35^ Groß, 2017;^12^ Heeg, 2019;^25^ Hillen, 2017a;^6^ Hillen, 2017b;^47^ Hillen, 2023;^29^ Kurian, 2017;^43^ Lehmann, 2020^51^/Lehmann, 2021;^23^* Lund, 2009;^37^ Lux, 2020;^58^ Lysack, 2013;^S2^ Meyer, 2015;^55^ Minamitani, 2021;^39^ Mordechai, 2015;^40^ Nagler, 2010;^36^ Oskay-Özcelik, 2007;^66^ Parikh, 2013;^42^ Philip, 2010;^13^ Pollack, 2015;^38^ Radhakrishnan, 2015;^32^ Rajan, 2021;^49^ Ramsey, 2011;^54^ Ruetters, 2016;^10^ Schook, 2014;^57^ Schulmeyer, 2023;^14^ Tam, 2005;^33^ van der Velden, 2021;^60^ Xu, 2011^41^ |
| 3 | | Country | Australia | 4 | Olver, 2020;^31^ Philip, 2010;^13^ Philip, 2011;^50^ Tattersall, 2009^1^ |
|  |  |  | China (Hong Kong) | 1 | Tam, 2005^33^ |
|  |  |  | France | 1 | Sanchez, 2021^44^ |
|  |  |  | Germany | 8 | Cecon, 2019;^68^ Fuchs, 2017;^30^ Groß, 2017;^12^ Könsgen, 2022;^59^ Loehberg, 2020;^46^ Lux, 2020;^58^ Oskay-Özcelik, 2007;^66^ Schulmeyer, 2023^14^ |
|  |  |  | India | 2 | Goud, 2021;^35^ Rajan, 2021^49^ |
|  |  |  | Israel | 1 | Mordechai, 2015^40^ |
|  |  |  | Italy | 1 | Maruzzo, 2021^7^ |
|  |  |  | Japan | 1 | Minamitani, 2021^39^ |
|  |  |  | The Netherlands | 7 | de Heus, 2021;^26^ Heeg, 2019;^25^ Hillen, 2018;^52^ Hillen, 2023;^29^ Lehmann, 2020^51^ /Lehmann, 2021;^23^* Schook, 2014;^57^ van der Velden, 2021^60^ |
|  |  |  | Switzerland | 1 | Peier-Ruser, 2018^67^ |
|  |  |  | Taiwan | 1 | Chang, 2013^11^ |
|  |  |  | USA | 23 | Al Achkar, 2022;^45^ Beer, 2022;^4^ Blazek, 2021;^22^ Chan & Epstein, 2005;^69^ Coffey, 2018;^53^ de Roo, 2020;^34^ Denberg, 2006;^48^ Garcia, 2018;^20^ Goldman, 2009;^27^ Hillen, 2017b;^47^ Khazai, 2015;^56^ Kurian, 2017;^43^ Lipitz-Snyderman, 2022;^17^ Lund, 2009;^37^ McCranie, 2024;^18^ Meyer, 2015;^55^ Nagler, 2010;^36^ Parikh, 2013;^42^ Pollack, 2015;^38^ Radhakrishnan, 2015;^32^ Ramsey, 2011;^54^ Weinfurtner, 2018;^S1^ Xu, 2011^41^ |
|  |  |  | International | 5 | Greenfield, 2021;^8^ Hillen, 2017a;^6^ Palmieri, 2017;^61^ Payne, 2014;^9^ Ruetters, 2016^10^ |
| 4 | | Place of residence | Metropolitan vs non-metropolitan | 2 | Goud, 2021;^35^ Mordechai, 2015;^40^ |
| 5 | | Patient age | Pediatric | 2 | Greenfield, 2021;^8^ Mordechai, 2015^40^ |
|  |  |  | Adult | 33 | Blazek, 2021;^22^ Cecon, 2019;^68^ de Roo, 2020;^34^ Goud, 2021;^35^ Greenfield, 2021;^8^ Groß, 2017;^12^ Heeg, 2019;^25^ Hillen, 2017a;^6^ Hillen, 2017b;^47^ Hillen, 2023;^29^ Lehmann, 2020^51^/Lehmann, 2021; ^23^* Lipitz-Snyderman, 2022;^17^ Loehberg, 2020;^46^ Lund, 2009;^37^ Lux, 2020;^58^ Lysack, 2013;^52^ McCranie, 2024;^18^ Meyer, 2015;^55^ Minamitani, 2021;^39^ Nagler, 2010;^36^ Olver, 2020;^31^ Oskay-Özcelik, 2007;^66^ Parikh, 2013;^42^ Peier-Ruser, 2018;^67^ Pollack, 2015;^38^ Radhakrishnan, 2015;^32^ Ramsey, 2011;^54^ Ruetters, 2016;^10^ Schook, 2014;^57^ Schulmeyer, 2023;^14^ Tam, 2005;^33^  Tattersall, 2009;^1^ van der Velden, 2021^60^ |
|  |  |  | Older adult (≥65 years) | 5 | Goud, 2021;^35^ Greenfield, 2021;^8^ Groß, 2017;^12^ Pollack, 2015;^38^ Radhakrishnan, 2015^32^ |
| 6 | | Patient gender^†^ | Male vs female | 6 | Fuchs, 2017;^30^ Greenfield, 2021;^8^ Hillen, 2017a;^6^ Payne, 2014;^9^ Schulmeyer, 2023;^14^ Tattersall, 2009^1^ |
| 7 | Patient educational attainment | | High school diploma vs degree/postgraduate degree | 12 | Cecon, 2019;^68^ Greenfield, 2021;^8^ Groß, 2017;^12^ Hillen, 2017a;^6^ Lund, 2009;^37^ Nagler, 2010;^36^ Olver, 2020;^31^ Parikh, 2013;^42^ Pollack, 2015;^38^ Radhakrishnan, 2017;^32^ Tam, 2005;^33^ Tattersall, 2009^1^ |
| 8 | Socioeconomic status | | Low vs high income | 7 | Blazek, 2021;^22^ Greenfield, 2021;^8^ Kurian, 2017;^43^ Lund, 2009;^37^ Mordechai, 2015;^40^ Pollack, 2015;^38^ Sanchez, 2021^44^ |
| 9 | Health insurance | | Yes/no | 5 | Blazek, 2021;^22^ Olver, 2020;^31^ Parikh, 2013;^42^ Pollack, 2015;^38^ Radhakrishnan, 2015^32^ |
| 10 | | Ethnicity | Black vs White | 2 | Pollack, 2015;^38^ Xu, 2011^41^ |
|  |  |  | Latina or Hispanic vs White | 1 | Parikh, 2013^42^ |

| 11 | Study type | Systematic reviews/meta-analyses | 5 | Greenfield 2021;^8^ Hillen, 2017a;^6^ Maruzzo, 2021;^7^ Payne, 2014;^9^ Ruetters, 2016^10^ |
| --- | --- | --- | --- | --- |
|  |  | Retrospective chart/imaging/ pathology reviews | 16 | Blazek, 2021;^22^ Chan & Epstein, 2005;^69^ Coffey, 2018;^53^ Coffey, 2017;^S3^ De Roo, 2020;^34^ Garcia, 2018;^20^ Goud, 2021;^35^ Heeg, 2019;^25^ Khazai, 2015;^56^ Lipitz-Snyderman, 2023;^17^ Lux, 2021;^58^ Lysack, 2013;^52^ Oskay-Ozcelik, 2007;^66^ Schook, 2014;^57^ Schulmeyer, 2023;^14^ Weinfurtner, 2018^S1^ |
|  |  | Cohort/cross-sectional surveys (quantitative) | 22 | Al Achkar, 2022;^45^ Beer, 2022;^4^ Cecon, 2019;^68^ Chang, 2013;^11^ De Heus, 2021;^26^ Denberg, 2006;^48^ Fuchs, 2017;^30^ Greenfield, 2012;^24^ Groß, 2017;^12^ Hillen, 2017b;^47^ Kurian, 2017;^43^ Lehmann, 2020^51^/Lehmann, 2021;^23^* Loehberg, 2020;^46^ Minamitani, 2021;^39^ Mordechai, 2015;^40^ Philip, 2010;^13‖^ Pollack, 2015;^38^ Radhakrishnan, 2017;^32^ Ramsey, 2011;^54^ Ruetters, 2016;^10^ Sanchez, 2021;^44^ Tam, 2005^33^ |
|  |  | Interviews, focus groups, narratives (qualitative) | 8 | Goldman, 2009;^27^ Hillen, 2018;^52§^ Nagler, 2010;^36^ Parikh, 2013;^42^ Peier-Ruser, 2018;^67^ Tattersall, 2009;^1^ Van der Velden, 2021;^60^ Xu, 2011^41^ |
|  |  | Policy/health services/economic evaluations | 6 | Lund, 2009;^37^ McCranie, 2024;^18^ Meyer, 2015;^55^ Olver, 2020;^31^ Pollack, 2015;^38^ Rajan, 2021^49^ |
|  |  | Longitudinal mixed-methods studies | 2 | Hillen, 2023;^29^ Philip, 2011^50§^ |
|  |  | Conceptual/narrative literature review | 1 | Moumjid, 2007^2^ |
| 12 | Study size (N) | <50 participants | 6 | Al Achkar, 2022;^45^ Denberg, 2006;^48^ Hillen, 2017b;^47^ Hillen, 2018;^52^ Peier-Ruser, 2018;^67^ Xu, 2011^41^ |
|  |  | ≥50–≤500 | 21 | Beer, 2022;^4^ Blazek, 2021;^22^ Cecon, 2019;^68^ Coffey, 2018;^53^ Coffey, 2017;^S3^ Fuchs, 2017;^30^ Garcia, 2018;^20^ Hillen, 2023;^29^ Lehmann, 2020^51^/Lehmann, 2021;^23^* Lipitz-Snyderman, 2022;^17^ Loehberg, 2020;^46^ Lux, 2020;^58^ Lysack, 2013;^S2^ McCranie, 2024;^18^ Mordechai, 2015;^40^ Olver, 2020;^31^ Philip, 2010;^13^ Ramsey, 2011;^54^ Schook, 2014;^57^ Tam, 2005;^33^ van der Velden, 2021^60^ |
|  |  | ≥500–≤1000 | 8 | Chan & Epstein, 2005;^69^ Heeg, 2019;^25^ Minamitani, 2021;^39^ Oskay-Özcelik, 2007;^66^ Parikh, 2013;^42^ Rajan, 2021;^49^ Schulmeyer, 2023;^14^ Weinfurtner, 2018^S1^ |
|  |  | ≥1000 | 14 | Chang, 2013;^11^ de Heus, 2021;^26^ de Roo, 2020;^34^ Goud, 2021;^35^ Groß, 2017;^12^ Khazai, 2015;^56^ Könsgen, 2022;^59^ Kurian, 2017;^43^ Lund, 2009;^37^ Meyer, 2015;^55^ Nagler, 2010;^36^ Pollack, 2015;^38^ Radhakrishnan, 2015;^32^ Tattersall, 2009^1^ |
| 13 | Cost-efficacy considered^¶^ |  | 2 | Moumjid, 2007;^2^ Palmieri, 2017^61^ |

*Lehmann, 2020^51^ and Lehmann, 2021^23^ are publications from the same mixed-methods study and are counted as one study in this analysis.
^†^Collated data on patient gender refers only to studies where both genders were compared and excludes studies in cancers only affecting one gender.
^‡^The Greenfield et al (2021)^8^ and Payne et al (2014)^9^ papers examined second-opinion seeking across medical specialities, including the oncology setting.
^§^Philip et al (2011)^50^ and Hillen et al (2018)^52^ evaluated the perspectives of oncologists on second medical opinions in cancer care; the studies did not include any patients with cancer.
^‖^Philip et al (2010)^13^ comprised parallel surveys of oncology patients with advanced cancer (n=52) and Australian medical oncologists (n=65).
^¶^These publications discussed cost-efficacy aspects briefly; neither of the reported studies provided full cost-efficacy analysis.

**Supplementary references**

**S1** Weinfurtner RJ, Niell B, Mekhail Y, et al Specialized second opinion interpretations of breast imaging: impact on additional workup and management. Clin Breast Cancer. 2018;18:e1031-e1036.

**S2** Lysack JT, Hoy M, Hudon ME, et al Impact of neuroradiologist second opinion on staging and management of head and neck cancer. J Otolaryngol Head Neck Surg. 2013;42:39.

**S3** Coffey K, D’Alessio D, Keating DM, et al Second-opinion review of breast imaging at a cancer center: is it worthwhile? AJR Am J Roentgenol. 2017;208:1386-1391.
